# A New Enterprise

**Published:** 1878-10

**Authors:** 


A NEW ENTERPRISE.—Wood's Library of Standard Medical Authors. For many years past Wm. Wood & Co., have had under consideration the feasibility of reproducing books, especially of foreign authors, in good style, and yet at prices greatly less than heretofore attempted. The high cost of labor, and of all the materials used in the manufacture of books, has been an insuperable obstacle. Even now it would be impossible to carry out systematically any such idea if the ordinary methods of trade were depended upon.

It is believed that the medical profession will welcome and generously sustain any well-directed effort of such character, and the following scheme has been prepared with much care, and is respectfully submitted for approval and support. They announce that in January, 1879, they will begin the publication of Medical Books by the most distinguished modern and standard authors, in monthly volumes of from 200 to 300 pages and upwards, handsomely and strongly bound, at the merely nominal price of One Dollar each. Estimating from the regular prices of the books so far selected for publication in 1879, subscribers to this Library will obtain about Fifty Dollars' worth of Medical Books for Twelve Dollars. Wood engravings and plates will be freely used whenever required. With the type and size of page adopted, it will frequently be possible to reproduce in one of these monthly volumes, an ordinary book of 400 to 500 pages, and costing from $4.00 to $6.00. They offer the following terms of sale: They say, "it would be impossible to publish books upon this plan without a very large assured sale for each volume. Wood's Library of Standard Medical Authors will, therefore, be sold by subscription only at Twelve Dollars a year. Payable invariably in advance. Subscriptions must be for a complete year. The volumes of this Library will not be sold separately."

